# Discovery of Natural Inhibitors of Cholinesterases from *Hydrangea*: In Vitro and In Silico Approaches

**DOI:** 10.3390/nu13010254

**Published:** 2021-01-17

**Authors:** Jayeong Hwang, Kumju Youn, Gyutae Lim, Jinhyuk Lee, Dong Hyun Kim, Mira Jun

**Affiliations:** 1 Department of Food Science and Nutrition, Dong-A University, Busan 49315, Korea; jyhwang18@donga.ac.kr (J.H.); kjyoun@dau.ac.kr (K.Y.); 2 Korean Bioinformation Center, Korea Research Institute of Bioscience and Biotechnology (KRIBB), Daejeon 34141, Korea; gyutae@kribb.re.kr (G.L.); jinhyuk@kribb.re.kr (J.L.); 3 Department of Bioinformatics, KRIBB School of Bioscience, Korea University of Sciences and Technology, Daejeon 34113, Korea; 4 Department of Medicinal Biotechnology, Dong-A University, Busan 49315, Korea; mose79@dau.ac.kr; 5 Department of Health Sciences, The Graduate School of Dong-A University, Busan 49315, Korea

**Keywords:** Alzheimer’s disease (AD), cholinesterase, molecular docking, thunberginol C, hydrangenol 8-*O*-glucoside pentaacetate

## Abstract

Alzheimer’s disease (AD) is a neurodegenerative disease conceptualized as a clinical-biological neurodegenerative construct where amyloid-beta pathophysiology is supposed to play a role. The loss of cognitive functions is mostly characterized by the rapid hydrolysis of acetylcholine by cholinesterases including acetylcholinesterase (AChE) and butyrylcholinesterase (BChE). Moreover, both enzymes are responsible for non-catalytic actions such as interacting with amyloid β peptide (Aβ) which further leads to promote senile plaque formation. In searching for a natural cholinesterase inhibitor, the present study focused on two isocoumarines from hydrangea, thunberginol C (TC) and hydrangenol 8-*O*-glucoside pentaacetate (HGP). Hydrangea-derived compounds were demonstrated to act as dual inhibitors of both AChE and BChE. Furthermore, the compounds exerted selective and non-competitive mode of inhibition via hydrophobic interaction with peripheral anionic site (PAS) of the enzymes. Overall results demonstrated that these natural hydrangea-derived compounds acted as selective dual inhibitors of AChE and BChE, which provides the possibility of potential source of new type of anti-cholinesterases with non-competitive binding property with PAS.

## 1. Introduction

Alzheimer’s disease (AD) is a neurodegenerative disease characterized by severe cognitive impairment [1]. Cholinergic deficit underlying the memory and cognitive decline is connected with decreased levels of the neurotransmitter, acetylcholine (ACh). Acetylcholinesterase (AChE), an important component of cholinergic synapses, is responsible for the hydrolysis of ACh [2]. The enzyme is found principally at neuromuscular junctions and cholinergic synapses in high concentrations. However, butyrylcholinesterase (BChE) is a non-specific type of cholinesterase involved in the hydrolysis of ACh, which is ubiquitously expressed in liver, blood serum, pancreas and associated with glial and endothelial cells in the brain [3]. Because of low expression in brain, the importance of BChE was underestimated in neurodegenerative diseases such as AD [4].

Along with AChE, BChE appears to be a co-regulator of ACh level in the brain [5]. In normal brain, AChE primarily hydrolyzes ACh while BChE plays only a supportive role. However, the level of AChE declined in the cortex and hippocampus of progressive AD patients, whereas that of BChE is substantially increased [6]. Furthermore, BChE maintained the cholinesterasic function in AChE knockout mouse models [7]. It is possible that these alterations are associated with the loss of cholinergic synapses and neurons, which demonstrates that the inhibitory action of both enzymes may become more significant as AD progresses [8].

Recent research has shown that AChE and BChE participate in AD progression. Amyloid-β peptide (Aβ), a major component of extracellular senile plaques, is a characteristic hallmark of AD [9]. Numerous studies demonstrated that Aβ fibrils are highly toxic causing additional downstream damage such as oxidative stress, mitochondrial dysfunction, calcium dyshomeostasis, inflammation and neuronal death. It is noteworthy that AChE accelerates Aβ assembly, where the peripheral binding site of AChE might be involved in amyloid fibril formation [10,11,12]. It was demonstrated that AChE constitutes a main co-factor in Aβ fibril complex, further leading to the conformational change of Aβ [13,14]. Moreover, these AChE-Aβ complexes have shown to be more neurotoxic than Aβ fibrils alone [12,15].

Although the role of BChE in AD is still not clear enough, several studies revealed the association of BChE and Aβ plaques that are of the fibrillar, β-sheet form of Aβ. Histochemical analysis of AD brain tissues indicated that BChE is present in Aβ plaques playing an important role in the subpopulation and maturation of Aβ plaques in transgenic AD mouse model [16]. Mesulam and Geula exhibited that BChE reactivity in Aβ plaque of demented brains was about five to six times higher than those of non-demented elderly ones [17]. BChE changed the life cycle of amyloid plaque by participating in the Aβ transformation from an initial benign to malignant form [18].

Cholinergic abnormalities have complex reciprocal interactions with other pathological hallmarks of AD including Aβ and tau. Previous study suggested that the cholinergic deficit triggered Aβ deposition and tau hyperphosphorylation in ways that contribute to the cognitive impairment [19]. Mori et al. demonstrated that anti-cholinesterase downregulated amyloidogenic and tau-generating pathways via stimulation of cholinergic receptors [20]. In addition, muscarinic acetylcholine receptor (mAhR) agonists have been associated with elevated soluble amyloid precursor protein α (sAPPα) release, suggesting that these agents activate a pathway that cleaves amyloid precursor protein (APP) within the Aβ domain and hence might prevent amyloid formation [21]. 

*Hydrangeae Dulcis Folium* (Hydrangea) has long been used as both traditional tea and medicine in the Asian countries, such as China, Japan and Korea [22]. The plant has been reported to possess anti-diabetic, anti-allergic and anti-bacterial activities [23,24,25,26,27]. The major active chemical constituents of the plant are isocoumarins, secoiridoids and stilbenes [28]. In searching for natural cholinesterase inhibitors, 50 natural plant extracts have been screened and Hydrangea displayed a potential inhibitory activity. The present study investigated the anti-cholinesterase effects of two major Hydrangea-derived compounds, thunberginol C (TC) and hydrangenol 8-*O*-glucoside pentaacetate (HGP), focusing on enzyme inhibition via both in vitro and in silico approaches.

## 2. Materials and Methods

### 2.1. Materials and Chemicals

TC (≥98% purity) and HGP (≥98% purity) were obtained from ChemFaces (Wuhan, China). Galantamine, 5,5’-dithiobis-(2-nitrobenzoic acid) (DTNB), AChE, BChE, trypsin, chymotrypsin, elastase and their substrates were obtained from Sigma-Aldrich (St. Louis, MO, USA). A beta-site APP cleaving enzyme 1 (BACE1) fluorescence resonance energy transfer (FRET) assay kit was purchased from Pan Vera (Madison, WI, USA).

### 2.2. Cholinesterases Inhibition Assay, Enzyme Selectivity and Kinetic Study

Cholinesterases, trypsin, chymotrypsin, elastase and BACE1, assays were performed according to the previous methods [29]. AChE, BChE, trypsin, chymotrypsin and elastase were assayed according to the manual depicted in the reference using ACh iodide, butyrylthiocholine chloride, N-benzoyl-L-Arg-pNA, N-benzoyl-L-Tyr-pNA and N-succinyl-Ala-Ala-Ala-pNA as substrates, respectively. BACE1 assay was achieved using a Rh-EVNLDAEFK-Quencher as a substrate. To prove the kinetic mechanisms of hydrangea-derived compounds towards cholinesterases, both Dixon plot and Lineweaver–Burk complementary methods were performed. The inhibitory constant (Ki) was obtained by Dixon plot, and Vmax and Km were defined by Lineweaver–Burk plots. These kinetic parameters were calculated using the SigmaPlot™ (version 12.3, Systat Software, Inc., San Jose, CA, USA) [30].

### 2.3. In Silico Docking Analysis

X-ray crystal structures of human AChE (PDB code: 4PQE) and BChE (PDB code: 1P0I) were inquired from the Protein Data Bank. Compound identification number (CID) of TC and HGP were obtained from PubChem (10333412 and 13962966). The Autodock Vina program version 1.1.2 (The Scripps Research Institute, San Diego, CA, USA) was used to conduct protein-ligand docking simulation.

### 2.4. Statistics

All results were representative of three independent experiments and expressed as the mean ± SD. All statistical analysis was performed using statistical analysis system (version 9.3, Cary, NC, USA). Duncan’s multiple range test was used to determine significant differences.

## 3. Results

### 3.1. Cholinesterase Inhibitory Activity of Hydrangea-Derived Compounds

**Figure 1:**
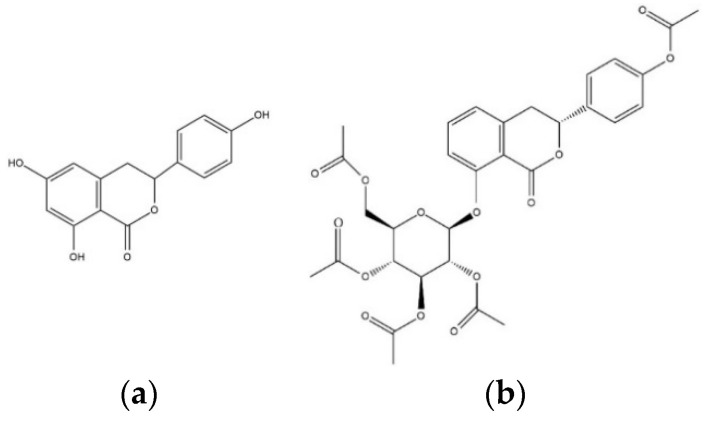
The chemical structures of (**a**) thunberginol C (TC) and (**b**) hydrangenol 8-*O*-glucoside pentaacetate (HGP).

To evaluate the ability to inhibit target enzymes, inhibitory effects of TC and HGP against in vitro AChE and BChE were determined (Table 1). The structures of the compounds were shown in Figure 1. TC and HGP had an IC_50_ value of 41.96 ± 1.06 µM and 22.66 ± 1.63 µM against AChE, respectively. In addition, two compounds showed anti-BChE activity with IC_50_ value of 42.36 ± 3.67 µM and 41.02 ± 3.03 µM, respectively.

Both AChE and BChE belong to a family of serine hydroxylases with sequence homology. To check the enzyme selectivity and specificity, the inhibitory activities on other serine proteases such as trypsin, chymotrypsin and elastase, and BACE1 were evaluated (Table 2). The results demonstrated that TC and HGP did not exhibit significant inhibition against serine proteases and BACE1, indicating that both compounds selectively inhibit AChE and BChE.

### 3.2. Evaluation of Inhibition Kinetics of Hydrangea-Derived Compounds

**Figure 2:**
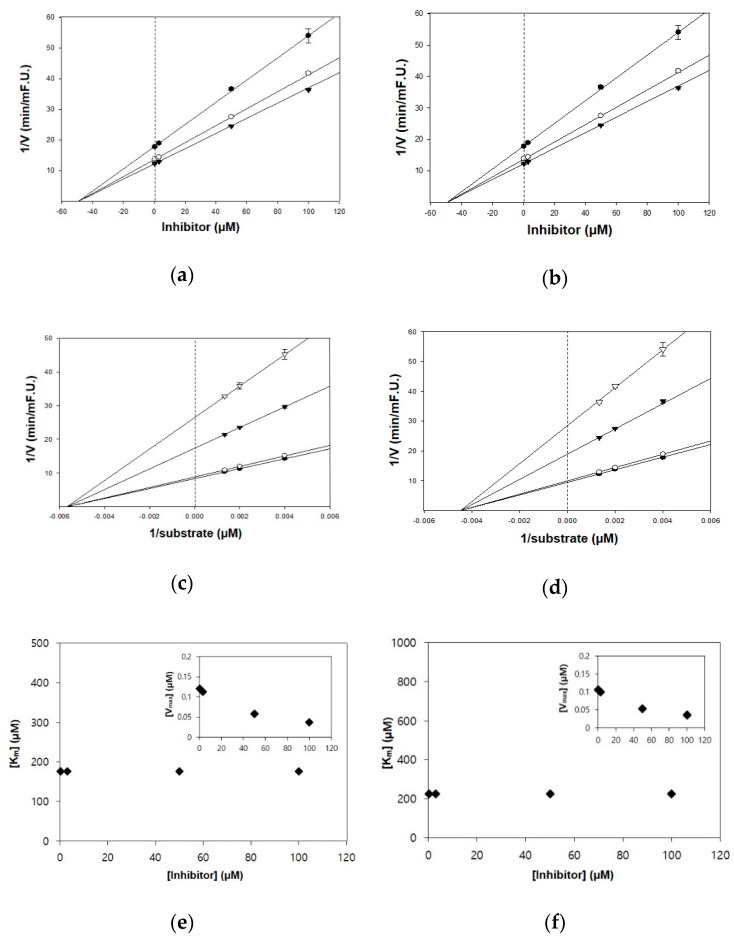
Kinetic plots of (**a**,**c**,**e**) acetylcholinesterase (AChE) and (**b**,**d**,**f**) butyrylcholinesterase (BChE) inhibition by TC. (**a**,**b**) In the Dixon plots, each symbol displays the substrate concentration: 250 µM (●); 500 µM (○); and 750 µM (▼). (**c**,**d**) In the Lineweaver–Burk plots, the concentrations of TC were as follows: 0.3 μM (●); 3 μM (○); 50 μM (▼); and 100 μM (▽). (**e**,**f**) Km values as a function of the concentrations of TC (Inset) dependence of the Vmax values on the inhibitor concentrations.

**Figure 3:**
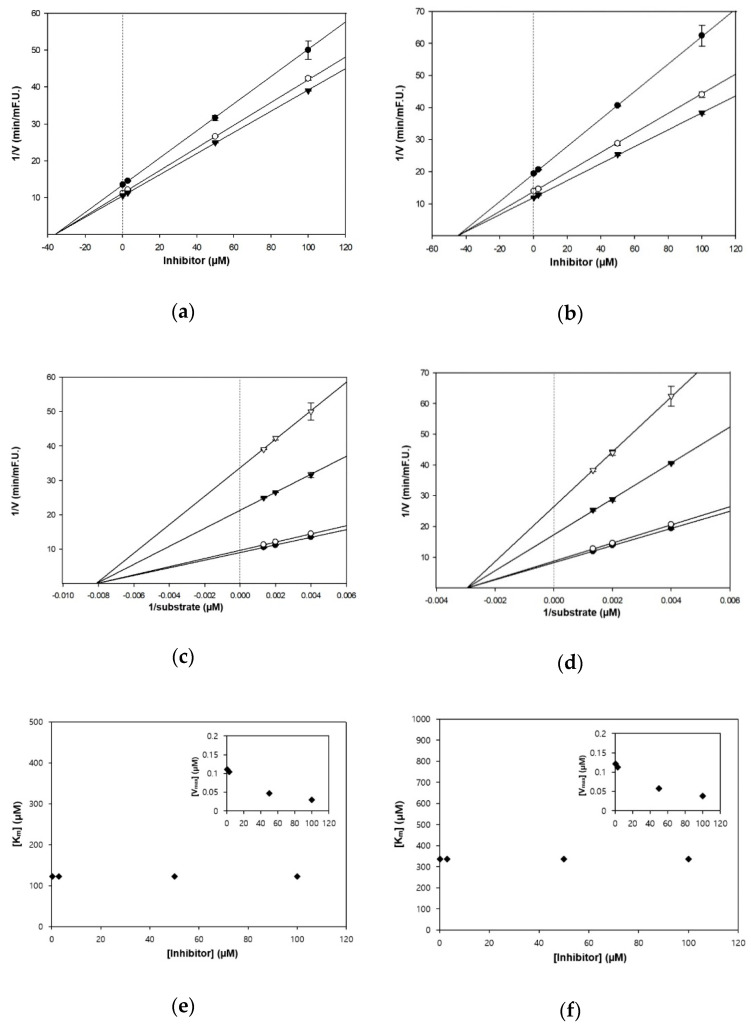
Kinetic plots of (**a**,**c**,**e**) AChE and (**b**,**d**,**f**) BChE inhibition by HGP. (**a**,**b**) In the Dixon plots, each symbol displays the substrate concentration: 250 µM (●); 500 µM (○); and 750 µM (▼). (**c**,**d**) In the Lineweaver–Burk plots, the concentrations of HGP were as follows: 0.3 μM (●); 3 μM (○); 50 μM (▼); and 100 μM (▽). (**e**,**f**) Km values as a function of the concentrations of HGP (Inset) dependence of the Vmax values on the inhibitor concentrations.

The kinetic studies of TC and HGP against cholinesterases were conducted with different concentrations of substrates and inhibitors (Table 1, Figure 2 and Figure 3). The Lineweaver–Burk plots exhibited that the intersections of fitting lines with various concentrations of TC and HGP were neither on the Y axis, suggesting that both compounds were non-competitive AChE inhibitors, with Ki values of 45.6 μM and 36.1 μM, respectively. Furthermore, the Ki values of two compounds against BChE were 49.2 µM and 44.9 µM, respectively. Since the Ki value shows the concentration needed to form an enzyme-inhibitor complex, lower Ki values may therefore, represent more effective cholinesterase inhibition, which is essential for the development of prevention candidates in AD.

### 3.3. Molecular Interaction Mechanism of Cholinesterases and Hydrangea-Derived Compounds

**Figure 4:**
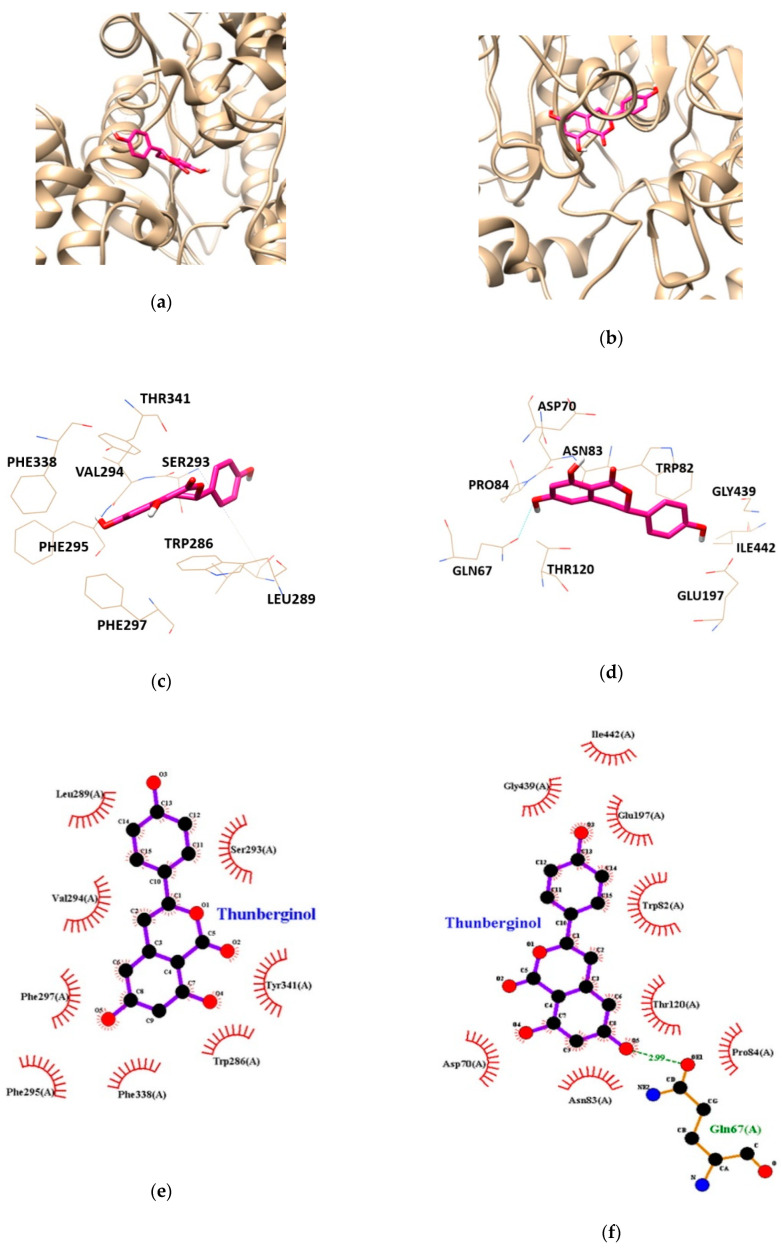
The best docking poses between (**a**) acetylcholinesterase (AChE), (**b**) butyrylcholinesterase (BChE) and thunberginol C (TC). Hydrogen and hydrophobic interaction diagram of (**c**,**e**) AChE and (**d**,**f**) BChE. TRP, tryptophan; LEU, leucine; SER, serine; VAL, valine; PHE, phenylalanine; THR, threonine; GLN, glutamine; ASP, aspartate; ASN, asparagine; PRO, proline; GLU, glutamate; GLY, glycine; ILE, isoleucine.

Molecular docking study was performed to gain insights into the targeted enzyme-inhibitor interaction and binding energy. AChE-TC and BChE-TC complexes had free energies of −6.78, and −7.87 kcal/mol, respectively (Table 3). As shown in Table 3 and Figure 4, the binding sites for AChE-TC complex were formed by van der Waals interaction with the residues TRP286, LEU289, SER293, VAL294, PHE295, PHE297, PHE338 and TYR341. BChE-TC complex formed by one hydrogen bond with interacting residues GLN67. In addition, BChE residues including ASP70, TRP82, ASN83, PRO84, THR120, GLU197, GLY439 and ILE442 were found to be responsible for hydrophobic interactions with TC. In particular, hydrophobic interaction was involved on both AChE and BChE applying with peripheral anionic site (PAS) residues of AChE-TRP286 and TRY341 and, BChE-ASP70, respectively, suggesting TC inhibits both cholinesterases activity in allosteric modes.

**Figure 5:**
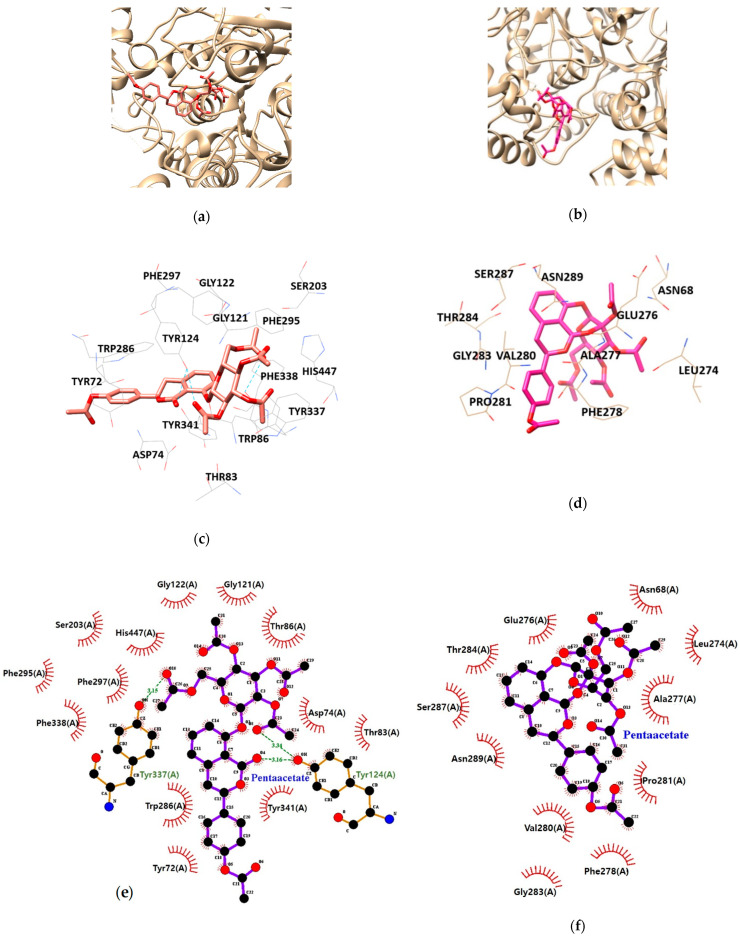
The best docking poses between (**a**) acetylcholinesterase (AChE), (**b**) butyrylcholinesterase (BChE) and hydrangenol 8-*O*-glucoside pentaacetate (HGP). Hydrogen and hydrophobic interaction diagram of (**c**,**e**) AChE and (**d**,**f**) BChE. TYR, tyrosine; ASP, aspartate; THR, threonine; TRP, tryptophan; GLY, glycine; SER, serine; PHE, phenylalanine; HIS, histidine; ASN, asparagine; LEU, leucine; GLU, glutamate; ALA, alanine; VAL, valine; PRO, proline.

As illustrated in Table 3 and Figure 5, HGP interacts with the residues TYR124, and TYR337 of AChE via hydrogen bond interaction. Moreover, hydrophobic interactions between HGP and 15 residues of AChE (TYR72, ASP74, THR83, TRP86, GLY121, GLY122, TYR124, SER203, PHE295, TRP286, PHE297, TYR337, TYR341, PHE338 and HIS447) were demonstrated to be crucial for binding to the PAS (TYR72, TYR124, TRP286, TYR337 and TYR341). In the formation of BChE-HGP complex, ASN68, LEU274, GLU276, ALA277, PHE278, THR284, VAL280, PRO281, SER287, GLY283 and ASN289 were involved in hydrophobic interactions with HGP.

Recently, kinetic studies for AChE propose the existence of two substrate-binding sites, the PAS, and catalytic active site (CAS). The PAS of AChE is composed of several aromatic residues including TRY72, TRY124, TRY337, TRY341 and TRP286, located at an entrance to the active site gorge. Tacrine, the first AChE inhibitor permitted by the FDA, interacted with the CAS of AChE. However, due to its side effects including acute liver toxicity, tacrine has been gradually withdrawn from the market [31]. To overcome this disadvantage, research on new type of AChE inhibitors has been elevated.

The PAS of AChE traps its substrate ACh and transfers it to the deep catalytic site, so when an inhibitor binds to PAS, it can block entry of the substrate into the gorge. In addition, this PAS site plays a crucial role in polymerization of Aβ, involving AChE-Aβ complexes, which accelerate fibril formation [12,15]. BChE also was found to associate with β-pleated sheets of amyloid fibrils [16,32].

The pathological hallmarks of AD have multifaceted and reciprocal interactions with the cholinergic lesion. Regarding the cholinergic-amyloid-axis hypothesis, several compelling evidences supported the synergistic interaction between cholinergic system and Aβ metabolism. Selective BChE inhibitor not only increased ACh level but also decreased that of Aβ in APP/presenilin (PS) transgenic mice [33]. Cisse et al. demonstrated that mAhR M1 agonists stimulated sAPPα release via protein kinase C (PKC)-dependent α-secretase activation without interrupting BACE1 [21]. Activation of mAhR decreased Aβ level by augmented α-secretase activity, implying APP process shift toward the non-amyloidogenic pathway [34]. Furthermore, activation of nicotinic acetylcholine receptor α7 attenuated Aβ-caused toxicity by upregulation of the phosphatidylinositol-3-kinase (PI3K)-protein kinase B (Akt) and downregulation of glycogen synthesis kinase-3 (GSK-3) [35]. Chu et al. demonstrated that reducing GSK-3 activity via PI3K/Akt signaling pathway prevented hyperphosphorylation of tau in transgenic mouse model of AD [36].

Natural compounds are considered as a potential source of new type of cholinesterase inhibitors due to their structural diversity, moderate to high biological activity and low toxicity. Several studies have been carried out toward discovery of natural anti-cholinesterase obtained from plants, fungus and marine organisms with IC_50_ values ranging from at a range from 2 to 200 µM [37,38]. Multi-targeted approach may provide better advantage than a single-targeted one for multifactorial diseases such as AD. The present study demonstrates the potential anti-cholinesterase property of TC and HGP as novel dual inhibitors of both AChE and BChE via non-competitive binding with PAS.

## 4. Conclusions

Hydrangea-derived compounds showed novel and dual inhibitory effect toward both AChE and BChE, that is selective and specific. In addition, kinetic and docking analysis demonstrated that TC and HGP act as non-competitive anti-cholinesterase through interaction with PAS of enzymes. This novel finding suggests that hydrangea-derived compounds may act an alternative symptomatic treatment for Alzheimer’s cognitive and behavioral symptoms and potentially associated with better clinical outcomes than available single-ligand inhibitors. Further and in-depth human study is required in near future.

## Figures and Tables

**Table 1:** Cholinesterases inhibitory activities, inhibition type, and dissociation constants (Ki) of TC and HGP.

Compounds	AChE			BChE		
	IC_50_^1^	Ki Value ^2^	Inhibition Type ^3^	IC_50_^1^	Ki Value ^2^	Inhibition Type ^3^
TC ^4^	41.96 ± 1.06	45.6	Non-competitive	42.36 ± 3.67	49.2	Non-competitive
HGP ^5^	22.66 ± 1.63	36.1	Non-competitive	41.02 ± 3.03	44.9	Non-competitive
Galantamine ^6^	1.72 ± 0.13	- ^7^	Competitive	12.21 ± 0.55	-	Competitive

^1^ IC_50_ (µM) was expressed as mean ± S.D. of triplicate experiments. ^2^ Ki value (µM) showed the binding affinity for enzyme-inhibitor complex. ^3^ Inhibition type of inhibitor determined by Dixon and Lineweaver–Burk plots. ^4^ HGP, hydrangenol 8-*O*-glucoside pentaacetate. ^5^ TC, thunberginol C. ^6^ Galantamine was used as a positive control in the cholinesterases assays. ^7^ - not tested.

**Table 2:** Inhibitory activities (%) ^1^ of TC and HGP against BACE1 and serine proteases.

Compounds (µM)		BACE1	Trypsin	Chymotrypsin	Elastase
TC	50	25.81 ± 3.11	−0.27 ± 1.51	6.04 ± 2.42	1.89 ± 1.09
	100	28.68 ± 1.87	0.07 ± 2.00	8.50 ± 1.40	0.42 ± 0.36
HGP	50	20.02 ± 0.52	1.26 ± 3.36	7.16 ± 3.38	3.77 ± 1.66
	100	25.21 ± 1.60	3.12 ± 1.69	5.15 ± 3.70	1.47 ± 1.31

^1^ The inhibitory activity (%) is expressed as mean ± SD of triplicate experiments.

**Table 3:** Binding interaction of TC and HGP with cholinesterases.

Enzymes	Ligands	Free Energy (kcal/mol)	No. of H-Bond	Residues	Bond Distance (Å)	van der Waals Residues
AChE	TC	−6.78	-			TRP286, LEU289, SER293, VAL294, PHE295, PHE297, PHE338, TYR341
	HGP	−9.77	3	TYR124, TYR337	3.16/3.34 3.15	TYR72, ASP74, THR83, TRP86, GLY121, GLY122, TYR124, SER203, TRP286, PHE295, PHE297, TYR337, TYR341, PHE338, HIS447
BChE	TC	−7.87	1	GLN67	2.99	ASP70, TRP82, ASN83, PRO84, THR120, GLU197, GLY439, ILE442
	HGP	−0.96				ASN68, LEU274, GLU276, ALA277, PHE278, THR284, VAL280, PRO281, SER287, GLY283, ASN289

AChE, aceylcholinesterase; BChE, butyrylcholinesterase; TC, thunberginol C; HGP, hydrangenol 8-*O*-glucoside pentaacetate; -, not detected; TYR, tyrosine; GLN, glutamine; TRP, tryptophan; LEU, leucine; SER, serine; VAL, valine; PHE, phenylalanine; ASP, aspartate; THR, threonine; GLY, glycine; HIS, histidine; ASN, asparagine; PRO, proline; GLU, glutamate; ILE, isoleucine.

## Data Availability

Not applicable.

## References

[1] Afshari A.R., Sadeghnia H.R., Mollazadeh H. (2016). A review on potential mechanisms of *Terminalia chebula* in Alzheimer’s disease. Adv. Pharmacol. Sci..

[2] Contestabile A. (2011). The history of the cholinergic hypothesis. Behav. Brain Res..

[3] Mushtaq G., Greig N.H., Khan J.A., Kamal M.A. (2014). Status of acetylcholinesterase and butyrylcholinesterase in Alzheimer’s disease and type 2 diabetes mellitus. CNS Neurol. Disord. Drug Targets.

[4] Li B., Stribley J.A., Ticu A., Xie W., Schopfer L.M., Hammond P., Brimijoin S., Hinrichs S.H., Lockridge O. (2000). Abundant tissue butyrylcholinesterase and its possible function in the acetylcholinesterase knockout mouse. J. Neurochem..

[5] Geula C., Darvesh S. (2004). Butyrylcholinesterase, Cholinergic Neurotransmission and the pathology of Alzheimer’s disease. Drugs Today (Barc.).

[6] Arendt T., Brückner M.K., Lange M., Volker B. (1992). Changes in acetylcholinesterase and butyrylcholinesterase in Alzheimer’s disease resemble embryonic development-a study of molecular forms. Neurochem. Int..

[7] Mesulam M.M., Guillozet A., Shaw P., Levey A., Duysen E.G., Lockridge O. (2002). Acetylcholinesterase knockouts establish central cholinergic pathways and can use butyrylcholinesterase to hydrolyze acetylcholine. Neuroscience.

[8] Ballard C.G., Greig N.H., Guillozet-Bongaarts A.L., Enz A., Darvesh S. (2005). Cholinesterases: Roles in the brain during health and disease. Curr. Alzheimer Res..

[9] Singh M., Kaur M., Kukreja H., Chugh R., Silakari O., Singh D. (2013). Acetylcholinesterase inhibitors as Alzheimer therapy: From nerve toxins to neuroprotection. Eur. J. Med. Chem..

[10] Bourne Y., Taylor P., Radić Z., Marchot P. (2003). Structural insights into ligand interactions at the acetylcholinesterase peripheral anionic site. EMBO J..

[11] Galdeano C., Viayna E., Arroyo P., Bidon-Chanal A., Blas J.R., Muñoz-Torrero D., Luque F.J. (2010). Structural determinants of the multifunctional profile of dual binding site acetylcholinesterase inhibitors as anti-Alzheimer agents. Curr. Pharm. Des..

[12] Rees T., Hammond P.I., Soreq H., Younkin S., Brimijoin S. (2003). Acetylcholinesterase promotes β-amyloid plaques in cerebral cortex. Neurobiol. Aging.

[13] Alvarez A., Opazo C., Alarcon R., Garrido J., Inestrosa N.C. (1997). Acetylcholinesterase promotes the aggregation of amyloid-β-peptide fragments by forming a complex with the growing fibrils. J. Mol. Biol..

[14] Carvajal F.J., Inestrosa N.C. (2011). Interactions of AChE with Aβ aggregates in Alzheimer’s brain: Therapeutic relevance of IDN 5706. Front. Mol. Neurosci..

[15] De Ferrari G.V., Canales M.A., Shin I., Weiner L.M., Silman I., Inestrosa N.C. (2001). A structural motif of acetylcholinesterase that promotes amyloid β-peptide fibril formation. Biochemistry.

[16] Darvesh S., Cash M.K., Reid G.A., Martin E., Mitnitski A., Geula C. (2012). Butyrylcholinesterase is associated with β-amyloid plaques in the transgenic APPSWE/PSEN1dE9 mouse model of Alzheimer disease. J. Neuropathol. Exp. Neurol..

[17] Mesulam M.M., Geula C. (1994). Butyrylcholinesterase reactivity differentiates the amyloid plaques of aging from those of dementia. Ann. Neurol..

[18] Guillozet A.L., Smiley J.F., Marsh D.C., Mesulam M.M. (1997). Butyrylcholinesterase in the life cycle of amyloid plaques. Ann. Neurol..

[19] Ramos-Rodriguez J.J., Pacheco-Herrero M., Thyssen D., Murillo-Carretero M.I., Berrocoso E., Spires-Jones T.L., Bacskai B.J., Garcia-Alloza M. (2013). Rapid beta-amyloid deposition and cognitive impairment after cholinergic denervation in APP/PS1 mice. J. Neuropathol. Exp. Neurol..

[20] Mori F., Lai C.C., Fusi F., Giacobini E. (1995). Cholinesterase inhibitors increase secretion of APPs in rat brain cortex. Neuroreport.

[21] Cisse M., Braun U., Leitges M., Fisher A., Pages G., Checler F., Vincent B. (2011). ERK1-independent α-secretase cut of β-amyloid precursor protein via M1 muscarinic receptors and PKCα/ε. Mol. Cell. Neurosci..

[22] Zehnter R., Gerlach H. (1995). Enantiodifferentiation in taste perception of the phyllodulcins. Tetrahedron Asymmetry.

[23] Zhang H., Matsuda H., Yamashita C., Nakamura S., Yoshikawa M. (2009). Hydrangeic acid from the processed leaves of Hydrangea macrophylla var. thunbergii as a new type of anti-diabetic compound. Eur. J. Pharmacol..

[24] Kurume A., Kamata Y., Yamashita M., Wang Q., Matsuda H., Yoshikawa M., Kawasaki I., Ohta S. (2008). Synthesis of 3-substituted isocoumarins and their inhibitory effects on degranulation of RBL-2H3 cells induced by antigen. Chem. Pharm. Bull..

[25] Matsuda H., Wang Q., Matsuhira K., Nakamura S., Yuan D., Yoshikawa M. (2008). Inhibitory effects of thunberginols A and B isolated from *Hydrangeae dulcis folium* on mRNA expression of cytokines and on activation of activator protein-1 in RBL-2H3 cells. Phytomedicine.

[26] Wang Q., Matsuda H., Matsuhira K., Nakamura S., Yuan D., Yoshikawa M. (2007). Inhibitory effects of thunberginols A, B, and F on degranulations and releases of TNF-α and IL-4 in RBL-2H3 cells. Biol. Pharm. Bull..

[27] Zhang H., Matsuda H., Kumahara A., Ito Y., Nakamura S., Yoshikawa M. (2007). New type of anti-diabetic compounds from the processed leaves of *Hydrangea macrophylla var. thunbergii* (*Hydrangeae Dulcis Folium*). Bioorg. Med. Chem. Lett..

[28] Hashimoto T., Tori M., Asakawa Y. (1987). Three dihydroisocoumarin glucosides from *Hydrangea macrophylla* subsp. *serrata*. Phytochemistry.

[29] Lee J., Jun M. (2019). Dual BACE1 and cholinesterase inhibitory effects of phlorotannins from *Ecklonia cava*-An in vitro and in silico study. Mar. Drugs.

[30] Lee S., Youn K., Lim G., Lee J., Jun M. (2018). In silico docking and in vitro spproaches towards BACE1 and cholinesterases inhibitory effect of citrus flavanones. Molecules.

[31] Harel M., Schalk I., Ehret-Sabatier L., Bouet F., Goeldner M., Hirth C., Axelsen P.H., Silman I., Sussman J.L. (1993). Quaternary ligand binding to aromatic residues in the active-site gorge of acetylcholinesterase. Proc. Natl. Acad. Sci. USA.

[32] Morán M.A., Mufson E.J., Gomez-Ramos P. (1993). Colocalization of cholinesterases with beta amyloid protein in aged and Alzheimer’s brains. Acta Neuropathol. (Berl.).

[33] Greig N.H., Utsuki T., Ingram D.K., Wang Y., Pepeu G., Scali C., Yu Q.S., Mamczarz J., Holloway H.W., Giordano T. (2005). Selective butyrylcholinesterase inhibition elevates brain acetylcholine, augments learning and lowers Alzheimer beta-amyloid peptide in rodent. Proc. Natl. Acad. Sci. USA.

[34] Welt T., Kulic L., Hoey S.E., McAfoose J., Späni C., Chadha A.S., Fisher A., Nitsch R.M. (2015). Acute effects of Muscarinic M1 receptor modulation on AβPP metabolism and Amyloid-β levels in vivo: A Microdialysis study. J. Alzheimers Dis..

[35] Beaulieu J.M. (2012). A role for Akt and glycogen synthase kinase-3 as integrators of dopamine and serotonin neurotransmission in mental health. J. Psychiatry Neurosci..

[36] Chu J., Lauretti E., Praticò D. (2017). Caspase-3-dependent cleavage of Akt modulates tau phosphorylation via GSK3β kinase: Implications for Alzheimer’s disease. Mol. Psychiatry.

[37] Dos Santos T.C., Gomes T.M., Pinto B.A.S., Camara A.L., Paes A.M.A. (2018). Naturally occurring anticholinesterases inhibitors and their potential use for Alzheimer’s disease therapy. Front. Pharmacol..

[38] Moodie L.W.K., Sepčić K., Turk T., Frange Ž.R., Svenson J. (2019). Natural cholinesterase inhibitors from marine organisms. Nat. Prod. Rep..

